# Factors Contributing to Dementia Caregiving and Clinical Trial Participation in an Ethnically Diverse Sample

**DOI:** 10.1093/geroni/igaf122.1099

**Published:** 2025-12-31

**Authors:** Miriam Jocelyn Rodriguez, Mariana Stavig, Bailey Gardner, Richard Holden, Malaz Boustani, Lilian Golzarri-Arroyo, Jordan Hill

**Affiliations:** Indiana University Bloomington, Bloomington, Indiana, United States; Indiana University Bloomington, Bloomington, Indiana, United States; Indiana University Bloomington, Bloomington, Indiana, United States; Indiana University Bloomington, Bloomington, Indiana, United States; Indiana University, Indianapolis, Indiana, United States; Indiana University Bloomington, Bloomington, Indiana, United States; Indiana University Bloomington, Bloomington, Indiana, United States

## Abstract

**Introduction:**

Informal caregiving of Alzheimer’s disease and related dementias (ADRD) is impacted by various factors including race/ethnicity, socioeconomic status (SES), and care recipient behavioral and psychological symptoms of dementia (BPSD). Caregiver-targeted interventions should consider these factors as well as the impact they have on clinical trial participation.

**Method:**

First, we examined recruitment methods in a racially/ethnically diverse sample. The call-to-consent ratio was examined for recruitment occurring via 1) “Cold Call” to patients from a health system registry, and 2) “Trusted Messenger” where research staff called patients referred from a memory care physician. Second, baseline data from a large-scale clinical trial was examined (N = 167) to identify differences among various demographic groups. One-way ANOVAs were conducted to examine differences in caregiver burden, self-efficacy, perceived social support, and care recipient BPSD, and the effects of SES on these variables. The sample consisted of White (n = 121), Black/African American (n = 35) and Hispanic/Latino (n = 11) caregivers.

**Results:**

Enrollment rate was 23.88% for participants recruited via the “trusted messenger” method and 1.18% for those recruited from “cold calls” (z = 15.11, p ≈ 0.0000). Hispanic participants reported less self-efficacy in obtaining respite (p=.018). No other significant differences across ethnic/racial groups were identified. Education level and employment status were significantly associated with caregiver burden (p=.017; p=.016), higher household income was associated to perceived social support (p<.001) and self-efficacy of controlling upsetting thoughts (p=.024).

**Conclusion:**

SES significantly impacts the caregiver experience, especially among Hispanics, and recruitment from a trusted source is effective for racial/ethnically diverse participation.

